# The pharmacokinetics and pharmacodynamics of cefquinome against *Streptococcus agalactiae* in a murine mastitis model

**DOI:** 10.1371/journal.pone.0278306

**Published:** 2023-01-25

**Authors:** Qingwen Yang, Chenghuan Zhang, Xuesong Liu, Longfei Zhang, Kang Yong, Qian Lv, Yi Zhang, Liang Chen, Peng Zhong, Yun Liu

**Affiliations:** 1 Laboratory of Veterinary Pharmacology, Department of Animal Science and Technology, Chongqing Three Gorges Vocational College, Chongqing, China; 2 Heilongjiang Key Laboratory for Laboratory Animals and Comparative Medicine, Department of Veterinary Surgery, College of Veterinary Medicine, Northeast Agricultural University, Harbin, China; 3 Laboratory of Veterinary Pharmacology, Branch of Animal Husbandry and Veterinary of Heilongjiang Academy of Agricultural Sciences, Qiqihar, China; 4 Heilongjiang Key Laboratory of Veterinary Medicine, Branch of Animal Husbandry and Veterinary of Heilongjiang Academy of Agricultural Sciences, Qiqihar, China; 5 College of Animal Science and Veterinary Medicine, Henan Institute of Science and Technology, Xinxiang, China; INRAE Centre Val de Loire: Institut National de Recherche pour l’Agriculture l’Alimentation et l’Environnement Centre Val de Loire, FRANCE

## Abstract

Cefquinome is a new generation cephalosporin that is effective in the treatment of mastitis in animals. In this study, we evaluated the associations between the specific pharmacokinetics and pharmacodynamics (PK/PD) of cefquinome and its antibacterial activity against *Streptococcus agalactiae* in a mouse model of mastitis. After a single intramammary dose of cefquinome (30, 60, 120, and 240 μg/mammary gland), the concentration of cefquinome in plasma was analysed by liquid chromatography with tandem mass spectrometry (HPLC/MS–MS). The PK parameters were calculated using a one-compartment first-order absorption model. Antibacterial activity was defined as the maximum change in the *S*. *agalactiae* population after each dose. An inhibitory sigmoid E_max_ model was used to evaluate the relationships between the PK/PD index values and antibacterial effects. The duration for which the concentration of the antibiotic (%T) remained above the minimum inhibitory concentration (MIC) was defined as the optimal PK/PD index for assessing antibacterial activity. The values of %T > MIC to reach 0.5-log_10_CFU/MG, 1-log_10_ CFU/MG and 2-log_10_ CFU/MG reductions were 31, 47, and 81%, respectively. When the PK/PD index %T > MIC of cefquinome was >81% *in vivo*, the density of the *Streptococcus agalactiae* was reduced by 2-log_10_. These findings provide a valuable understanding to optimise the dose regimens of cefquinome in the treatment of *S*. *agalactiae* infections.

## Introduction

Bovine mastitis (BM) is an inflammatory condition of the mammary gland that is caused by trauma or infection and results in the reduced production of both casein proteins and milk [1]. BM is a major threat to the dairy industry as it can reduce the quality and volume of milk production. Acute mastitis damages the milk-secreting alveolar cells leading to a permanent reduction in milk yield [2].

*Streptococcus agalactiae* is a highly contagious obligate pathogenic bacterium found in bovine mammary glands (MGs) [3]. Before the widespread use of antibiotics, it was reported that >85% of BM cases were caused by *S*. *agalactiae* [4] due to low-grade persistent infections that do not spontaneously resolve. Unidentified infected cows can be reservoirs of infection when they remain untreated or segregated [5]. *S*.*agalactiae* is a fundamental threat to the dairy industry in many countries [6] and infection is associated with elevated bulk tank somatic cell counts (SCC) and standard plate counts. Economic losses resulting from *S*. *agalactiae* infections are due to production losses associated with bulk tank SCC, milk quality penalties associated with bulk tank SCC and standard plate counts, and decreases in milk quality associated with bulk tank SCC [7]. The prevalence of *S*. *agalactiae* in the 4 regions of China ranges between 5.3–17% and is highest in eastern China (17%). The subregional prevalence of *S*. *agalactiae* ranges between 2.0–25.0% and is highest in the Jiangsu province of eastern China [8]. Between 2017 and 2019, the prevalence of *S*. *agalactiae* in several regions of the Sichuan province was 33.6% [9]. The detection rate of *S*. *agalactiae* in Chinese bulk tank milk samples was 92.2% [10].

The widespread misuse of antibiotics has resulted in increased antibiotic resistance leading to treatment failure. Previous studies have shown that in 11 provinces of China, all of the *S*. *agalgcitae* strains collected from mastitic raw milk samples were multi-resistant to three or more antimicrobial agents [11]. Also, in inner Mongolia, the isolates of *S*. *agalactiae* from dairy cows with mastitis were found to have a high frequency of β-lactam resistance alone and with tetracyclin or erythromycin [12]. These data highlight the need to optimise the dosing regimens of antibiotics to produce maximum efficacy during treatment.

The pharmacokinetic/pharmacodynamic (PK/PD) integration model is an important method of dosage optimisation, particularly in animal infection models. Only a few studies have used dairy cows as experimental animals because of the management challenges and costs associated with feeding. Other domestic animals, such as goats and sheep, have been used as substitutes yet the cost of these models remains considerable [13]. In comparison, the use of murine models are highly advantageous. A murine mastitis model was first described by Chandler and has since been widely used to study BM [14–16]. Data from murine mastitis models should be interpreted with an appreciation of the differences between the MGs of mice and cows. Several studies have reported on the similarities of bacterial inoculation in the mouse and cow MGs in terms of PMN infiltration and tissue damage [13, 17].

Cefquinome is a newly developed cephalosporin-specific drug that is used in animals [18]. Cefquinome has good PKs and can be absorbed quickly to peak concentrations in the blood [19]. It accumulates at high concentrations in the lungs and MGs, and has low toxicity in animals making it a highly attractive treatment option for various bacterial infections [20, 21]. Compared to systemic therapy, the intramammary administration of cefquinome is more effective in eliminating the causative pathogens of BM [22]. However, no previous studies have investigated the association between the PK/PD indexes and the antibacterial activity of cefquinome in the treatment of *S*. *agalactiae* infections.

In this study, we used a mouse mastitis model to study the PK/PD integration of cefquinome against *S*. *agalactiae*. This approach was used to determine the most suitable PK/PD indexe of cefquinome for the treatment of *S*. *agalactiae* mastitis, and to obtain specific PK/PD index values under different antibacterial conditions.These data provide a valuable understanding of the dose optimisation of cefquinome in the treatment of *S*. *agalactiae* infections.

## Materials and methods

### Drugs, bacteria, and animals

Cefquinome sterile powder was obtained from Dr. Ehrenstorfer (lot number G130285; Augsburg, Germany). *S*. *agalactiae* 3–64 were isolated from dairy cows infected with mastitis. Kunming mice were purchased from the Hunan Silaike Jingda Laboratory Animal (Hunan, China). The mice were maintained in compliance with the American Association for Accreditation of Laboratory Animal Care guidelines [23]. All animal studies were approved by the Laboratory Animal Welfare and Ethics Committee of the Northeast Agricultural University (NEAUEC20191011).

### Analysis of minimum inhibitory concentration (MIC), minimum bactericidal concentration (MBC) and mutant prevention concentration (MPC)

MICs were determined by microdilution in compliance with the Clinical Laboratory Standards Institute guidelines [24]. Briefly, colonies were transferred into MHB supplemented with 5% mouse serum and incubated at 37°C on a shaking incubator (220 rpm). The final count was approximately 1×10^8^ CFU/mL. 10 μL (1×10^6^ CFU/mL) of the culture was used to inoculate each well of a 96-well plate containing broth with different concentrations of cefquinome. A series of two-fold dilutions was achieved by adding 100 μL culture aliquots to a 96-well plate. The MIC was considered the lowest concentration of cefquinome that inhibited bacterial growth in broth after 24 hrs incubation. MBC was determined using a single set of doubling dilutions. The MIC well and four other wells with drug concentrations higher than the MIC were used to establish the MBC using the spot plate count method. The lowest drug concentration that reduced the bacterial count by 99.9% of the original count after 18 hrs was defined as the MBC [25]. Mutant prevention concentrations were determined by applying a high count bacterial suspension (1.5×10^11^ colony-forming units (CFU)/mL) on to an agar plate containing different drug concentrations (1, 2, 4, 8, 16, 32, 64 and 128 multiples of the MIC for each isolate). The concentration ranges were narrowed down. The plates were incubated at 37°C for 72 h and checked for bacterial growth every 24 h. The MPC was defined as the lowest concentration of cefquinome that completely inhibited bacterial growth after 72-hrs [26]. All susceptibility tests were repeated in triplicates.

### Establishment of an LC-ESI-MS/MS method for the analysis of cefquinome

The plasma concentrations of Cefquinome were determined by LC-ESI-MS/MS as described previously [27]. Briefly, 100 μl of water containing 0.1% (v/v) formic acid and 100 μL of plasma were combined and vortexed for 3 min. The samples were then centrifuged at 5000 × g for 15 min and the supernatants were harvested. 20 μL of the supernatant was injected into the HPLC system. The limit of detection (LOD) and limit of quantification (LOQ) values for this assay were 0.005 and 0.01 μg/ml, respectively. The recoveries of cefquinome in the plasma samples were >85%. All inter- and intra-assay variations were measured by calculating the relative standard deviation (%RSD) and ensuring that it was <10%.

### *In vitro* killing curve analyses

After 6 hrs of culture, logarithmic phase *S*. *agalactiae* 3–64 were added to 10 mL of MH broth (5% foetal calf serum) and diluted to 10^6^ CFU/mL and 10^7^ CFU/mL. A series of concentrations of cefquinome (0×, 0.5×, 1×, 2×, 4×, 8×, and 16× MIC) were added to the bacterial suspensions which were then cultured and incubated at 37°C. The bacterial population was measured at 0, 3, 6, 9, and 12 hrs. After serial 10-fold dilution, samples were plated on to trypticase soy agar (TSA) plates (5% defibrinated sheep blood) and cultured for 18–20 hrs. The detection limit was 300 CFU/mL.

### Establishment of a murine mastitis model

A murine mastitis model was established based on previous studies [28, 29]. 8–12 day old pups were removed and lactating mice were anaesthetised with 1% pentobarbital sodium delivered by i.p injection. After 0.15 hrs, the L4 (4^th^ on the left) and R4 (4^th^ on the right) abdominal MGs were disinfected with 75% ethanol and the teat tip was cut using scissors. To prevent environmental bacterial contamination, <1 mm of the tissue was removed. Each teat was held with fine forceps and the duct orifice was located. 100 μL of *S*. *agalactiae* (5.2×10^5^ CFU/mL) was slowly injected through the orifice using a syringe with a blunt needle (<30 gauge).

### *In vivo* antibacterial efficacy

After *S*. *agalactiae* was injected into the mice, four mice were euthanized by CO_2_ asphyxiation and MG samples were collected at 3, 6, 9, 12, 24, 48, and 72 h. MG samples were homogenized and the visible bacterial colonies were counted to establish *in vivo* bacterial growth curves for four experimental groups and one control group. In the mastitis model, a single dose of cefquinome was administered to the MG at a range of concentrations (30, 60, 120, or 240 μg/gland). The control group was treated with saline solution. The limit of detection for the bacteria was 300 CFU/MG.

### The pharmacokinetics of cefquinome in murine plasma

PK experiments were performed on lactating Kunming mice. The mice were randomized into four experimental groups (n = 6 each). Sedation and analgesia management were performed as described by Zeng et al. [30]. Briefly, mice were added to an induction chamber (oxygen flow rate = 0.5–1.0 L/min). At the same time, 3%–5% of isoflurane vapour was applied for induction and then reduced to 1%–3% for maintenance. As stated above, after intramammary administration (30, 60, 120, and 240 μg/MG), retro-orbital blood samples (200 μL at each time point) were harvested at 0.083, 0.167, 0.25, 0.5, 0.75, 1, 2, 4, 6, 8, 10, and 12 h after cefquinome administration. The plasma samples were isolated by centrifugation for 10 min (2500 × *g*, 4°C). The supernatants were stored at -20°C for 2 weeks and the plasma cefquinome concentration was established via HPLC-MS/MS. The linearity of cefquinome quantitation was from 0.01–5 μg/mL and R^2^ was >0.99. Cefquinome extraction recovery in the plasma was >80%, and the coefficient of variation was <10% within and between runs. The respective limitations of quantification and detection were 0.01 μg/mL and 0.005 μg/mL. The main PK parameters were harvested using WinNonlin version 5.2.1 (Pharsight, MO, USA). These parameters were the half-life of first-order elimination (T_1/2e_), the half-life of absorption (T_1/2α_), peak plasma concentration (C_max_),and time of maximum plasma concentration (T_max_).

### PK/PD integration

The PK/PD indexes comprised the AUC/MIC (area under the time-concentration curve divided by MIC), %T > MIC (the percentage time for which the drug concentration exceeded MIC), and C_max_/MIC (peak concentration divided by MIC). The relationship between *in vivo* antibacterial effects (△log CFU/MG) and the PK/PD indexes were described using an inhibitory sigmoid E_max_ model [31, 32].


$$E=Emax-\frac{(Emax-E0)\times C_{e}^{N}}{{EC}_{50}^{N}+C_{e}^{N}}$$

where E denotes the antibacterial effect determined based on the maximum change in the bacterial counts (log_10_ CFU/MG) during 72 h after treatment; E_max_ indicates the maximum change in the bacterial counts in the control group; E_0_ represents the maximum change in the bacterial counts in the various experimental groups; EC_50_ is the PK/PD index values that produced antibacterial effects equal to 50% of the maximum; C_e_ is the PK/PD index; and N is the Hill coefficient, corresponding to the steepness of the effect curve associated with each of the PK/PD indexes.

### Statistical analyses

Statistical analyses were conducted by the analysis of variance. Significant differences in the data were analysed using Bonferroni correction and with a P-value threshold of <0.05 set for statistical significance [33].

## Results

### Chromatogram of cefquinome

**Fig 1:**
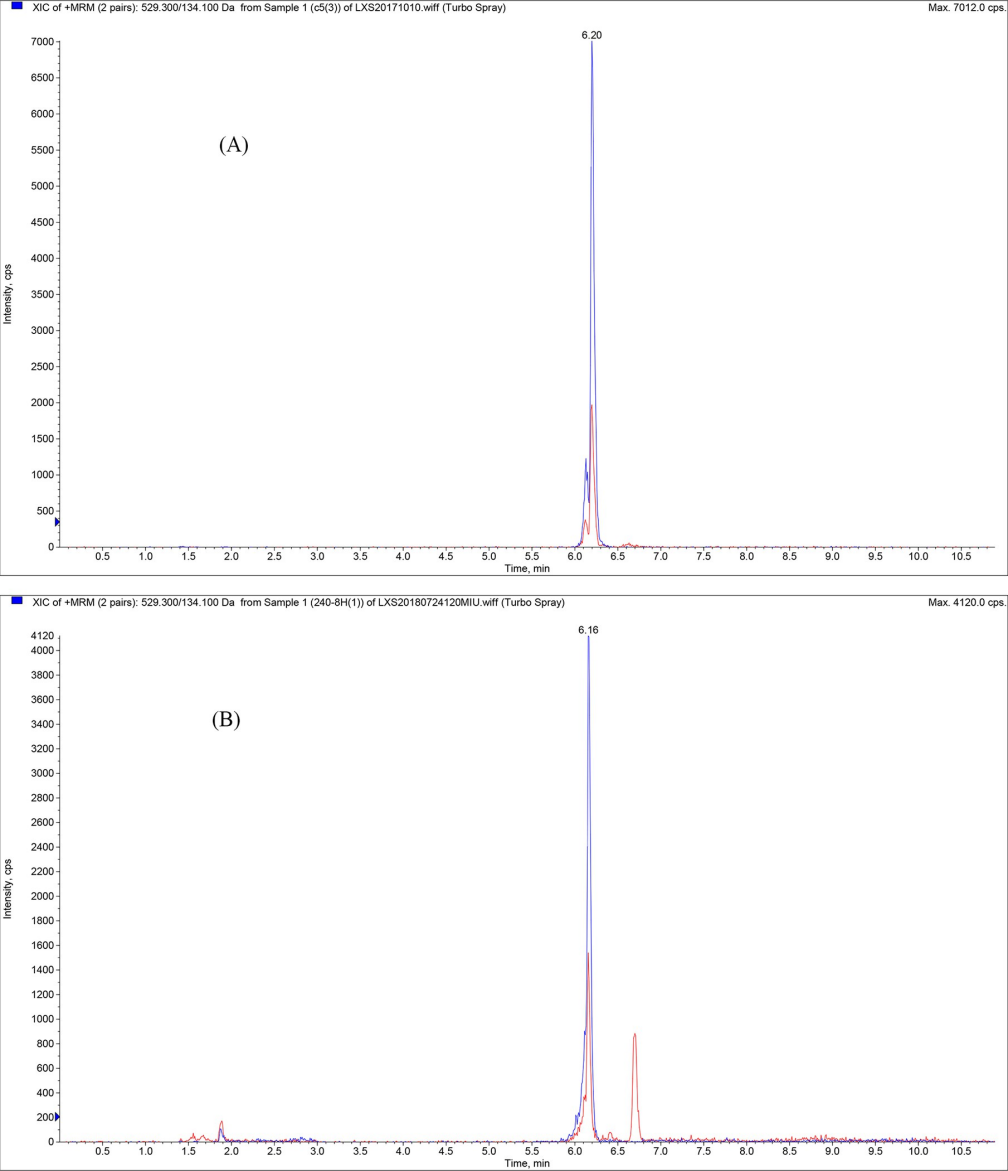
The standard cefquinome and test sample chromatograms. A sample of cefquinome (A) or test sample (B) was analyzed by LC-ESI-MS/MS. For the test sample, cefquinome was extracted as described in the method section and analyzed by LC-ESI-MS/MS (B).

The chromatograms for cefquinome in a cefquinome standard solution and the experimental samples are shown in Fig 1. This method had good specificity and was used for the determination of cefquinome.

### MIC, MCB and MPC

The MIC of cefquinome against *S*. *agalactiae* 3–64 was 0.03 μg/mL, the MBC was 0.06 μg/mL, and the MPC was 0.24 μg/mL. For the quality-control ATCC25922 (*Escherichia coli)* and ATCC29213 (*Staphylococcus aureus)* strains, the MIC values were 0.06 and 0.5 μg/mL, respectively.

### The PKs of cefquinome in murine plasma

**Fig 2:**
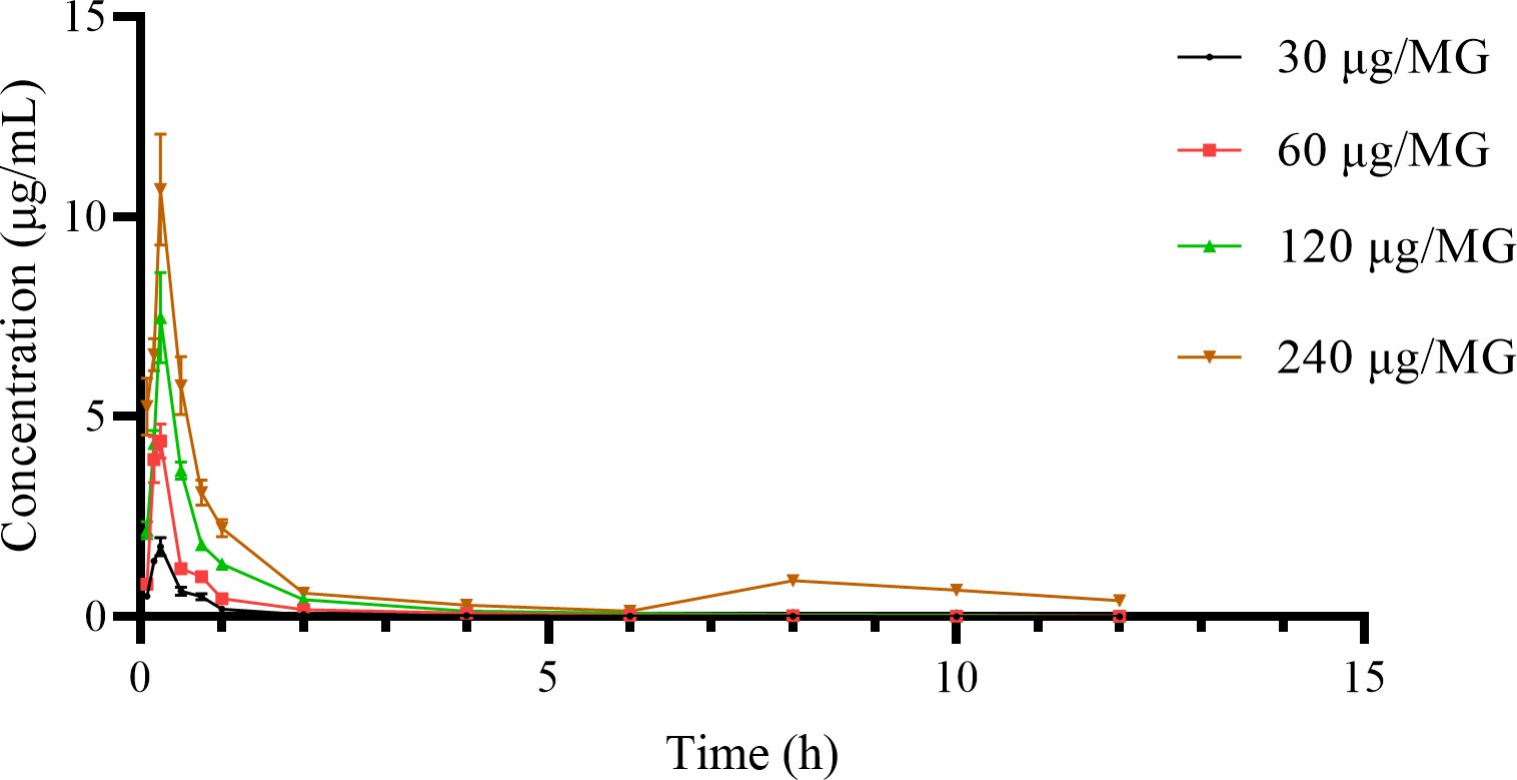
Plot of plasma concentrations of cefquinome versus time in a mouse model of *S*. ***agalactiae* mastitis (n = 6) following a single intramammary administration of 30 μg/MG, 60 μg/MG, 120 μg/MG, and 240 μg/MG.** The bars represent the standard deviations. The cefquinome concentration in plasma was determined by LC-ESI-MS/MS as described in the method section.

Due to the low protein binding (8%) [34] and precipitation in the samples, we believe that the cefquinome was almost entirely unbound in plasma. The concentration-time data are shown in Table 1. The cefquinome concentration-time curves for the different doses were generated (Fig 2). A one-compartment model with first-order absorption was fitted to calculate the PK parameters (Table 2). The time of the maximum plasma concentration (T_max_) was 0.20–0.25 hrs (mean, 0.22 hrs). The half-life of first-order elimination (T_1/2e_) was 0.47–0.69 hrs (mean, 0.65 hrs). The peak plasma concentration (C_max_) increased proportionately with increasing doses of cefquinome along with the area under the time-concentration curve (AUC).

**Table 1:** The cefquinome concentration (μg/mL) in the plasma of mice with *Streptococcus agalactiae-*induced mastitis following intramammary administration of 30, 60, 120, 240 μg/MG.

Time (h)	Does regimen (μg/MG)			
	30	60	120	240
0.083	0.52 ± 0.065	0.825 ± 0.113	2.17 ± 0.217	5.26 ± 0.273
0.167	1.389 ± 0.045	3.93 ± 0.143	4.33 ± 0.282	6.56 ± 0.398
0.25	1.755 ± 0.064	4.39 ± 0.351	7.48 ± 0.553	10.68 ± 0.49
0.5	0.641 ± 0.107	1.21 ± 0.102	3.66 ± 0.222	5.78 ± 0.602
0.75	0.504 ± 0.072	1.007 ± 0.136	1.81 ± 0.104	3.11 ± 0.321
1	0.196 ± 0.029	0.451 ±0.084	1.32 ±0.079	2.22 ± 0.164
2	0.054 ± 0.006	0.176 ± 0.036	0.375 ± 0.039	0.596 ± 0.047
4	0.029 ± 0.006	0.096 ± 0.023	0.143 ± 0.023	0.274 ± 0.043
6	0.019 ± 0.004	0.049 ± 0.01	0.093 ± 0.012	0.131 ± 0.013
8	0.014 ± 0.002	0.03 ± 0.003	0.054 ± 0.006	0.079 ± 0.008
10	0.011 ± 0.002	0.022 ± 0.003	0.038 ± 0.004	0.058 ± 0.006
12	0.009 ± 0.002	0.014 ± 0.002	0.021 ± 0.004	0.036 ± 0.004

**Table 2:** The pharmacokinetic parameters of cefquinome in the plasma of mice with *Streptococcus agalactiae-*induced mastitis.

Parameter (units)	Dosage (μg/MG)			
	30	60	120	240
T1/2_α_ (h)	0.08	0.07	0.07	0.07
T1/2_e_ (h)	0.61	0.51	0.38	0.46
T_max_ (h)	0.22	0.20	0.25	0.20
AUC (μg.h/mL)	1.16	2.39	5.41	8.33
C_max_ (μg/mL)	1.31	3.20	6.79	8.43
MRT (h)	0.84	0.78	1.06	0.98
Vss (L/kg)	0.22	0.18	0.23	0.28

T_1/2α_ indicates the absorption half-life; T_1/2e_ represents the elimination half-life; T_max_ denotes the time to achieve the maximum concentration; the AUC represents the area under the concentration-time curve; C_max_ indicates the maximum plasma concentration; MRT indicates the mean residence time; Vss represents the volume of distribution.

### *In vitro* killing curves

**Fig 3:**
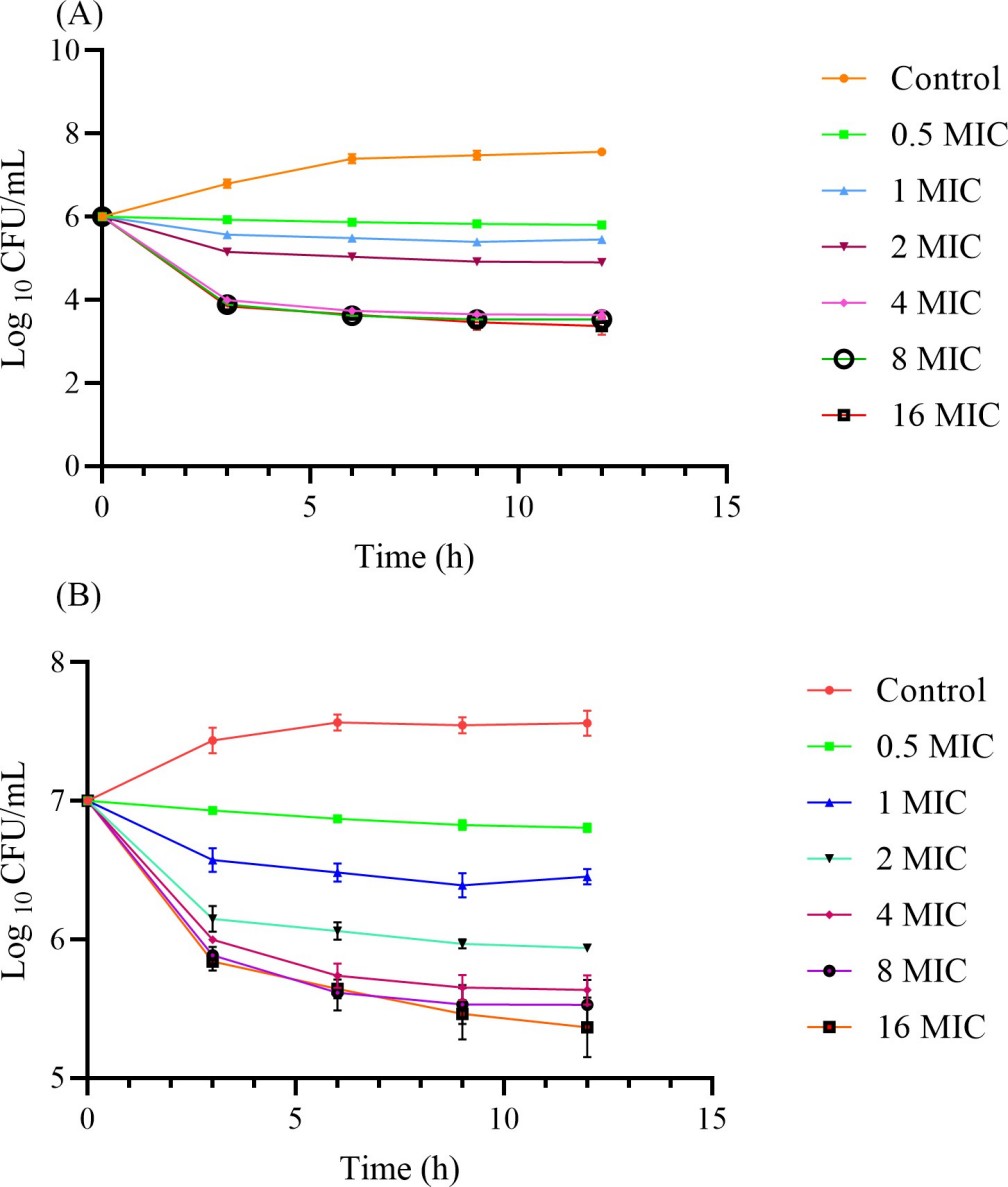
*In vitro* cefquinome killing curve against *S*. *agalactiae* 3–64. (A) Antibacterial effects begin at an initial inoculum of 10^6^ CFU/mL. (B) Antibacterial effects begin at an initial inoculum of 10^7^ CFU/mL. The bacterial population was measured at 0, 3, 6, 9, and 12 hrs by counting visible bacterial colonies.

The *in vitro* killing curves are presented in Fig 3 and show that cefquinome is a classical time-dependent drug. The killing rate and bactericidal effects did not increase with an increase in drug concentration. In the low-concentration group (< 4 × MIC), cefquinome did not have a bactericidal effect. When the concentration reached >4 × MIC, in the low-concentration-pathogens group, the maximum bactericidal effect achieved a 3-log_10_ CFU/mL reduction. However, in the high-concentration-pathogens group, the maximum bactericidal effect only achieved a 1.2-log_10_ CFU/mL reduction.

### *In vivo* antibacterial effect

**Fig 4:**
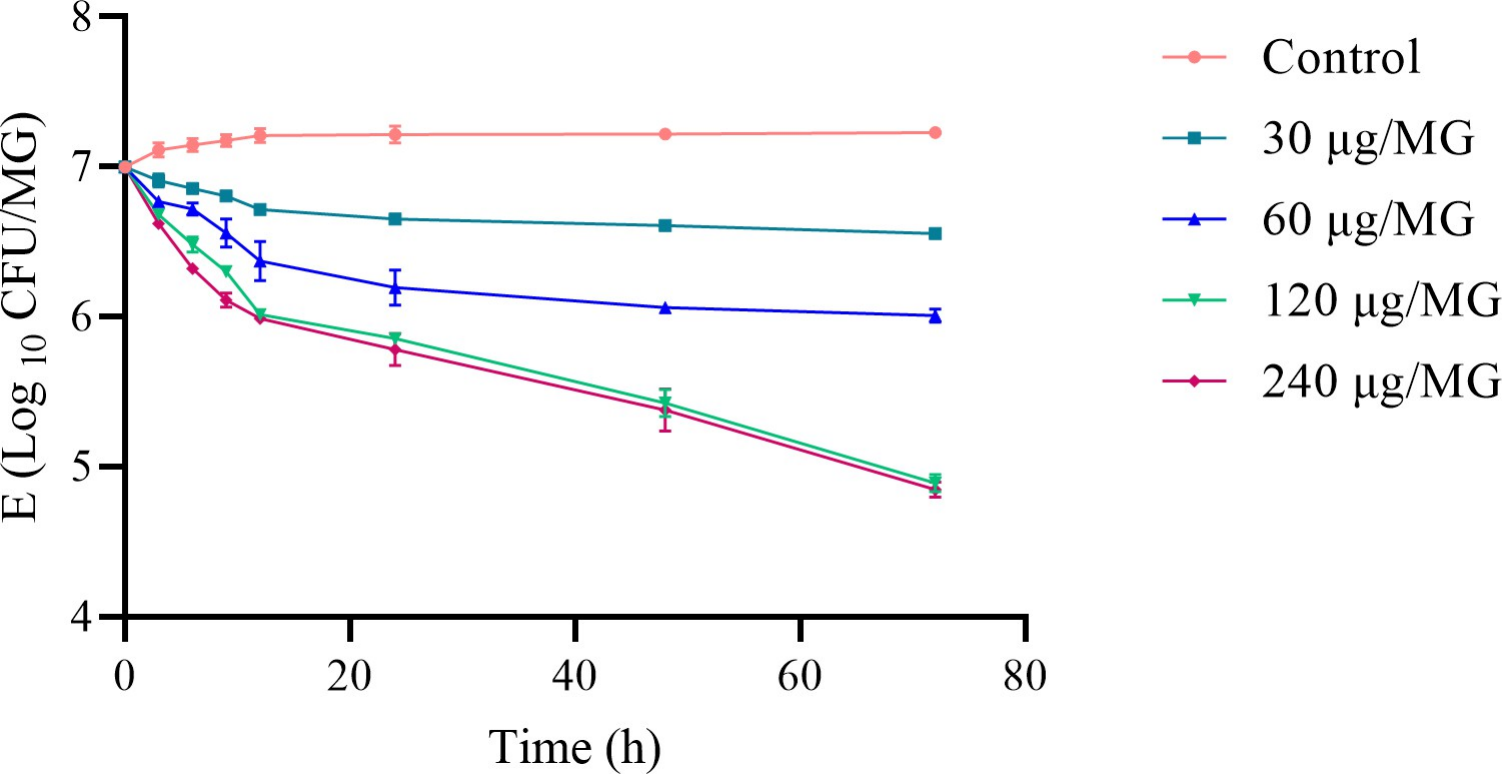
*In vivo* cefquinome PD studies in a murine mastitis model. The change in the log_10_ CFU/MG was measured after 72 hrs of treatment. Changes in the viable cell density (CFU/MG) of *S*. *agalactiae* and the concentrations of antibiotics (×MIC) *in vivo* following a single treatment with cefquinome. Test dosage regimens were a single dose of 30 μg/MG, 60 μg/MG, 120 μg/MG, and 240 μg/MG by intramammary administration, (n = 4 for mice, and 8 mammary glands). The bacterial population was measured by counting visible bacterial colonies.

The *in vivo* killing curves are shown in Fig 4. The killing rate of cefquinome in mice was lower than that observed in Mueller–Hinton Broth. However, the killing curve of cefquinome against *S*. *agalactiae* showed a classical time-dependent pattern. The 30, 60, 120, and 240 μg/MG experimental groups achieved 1.1-log_10_, 1.2-log_10_, 2.5-log_10_, and 2.8-log_10_CFU/MG reductions, respectively at 72 h. The 120 μg/gland and 240 μg/gland experimental groups almost achieved bactericidal effects but these did not change significantly within increasing drug concentration increased (*P>*0.05).

### PK/PD integration and analysis

**Fig 5:**
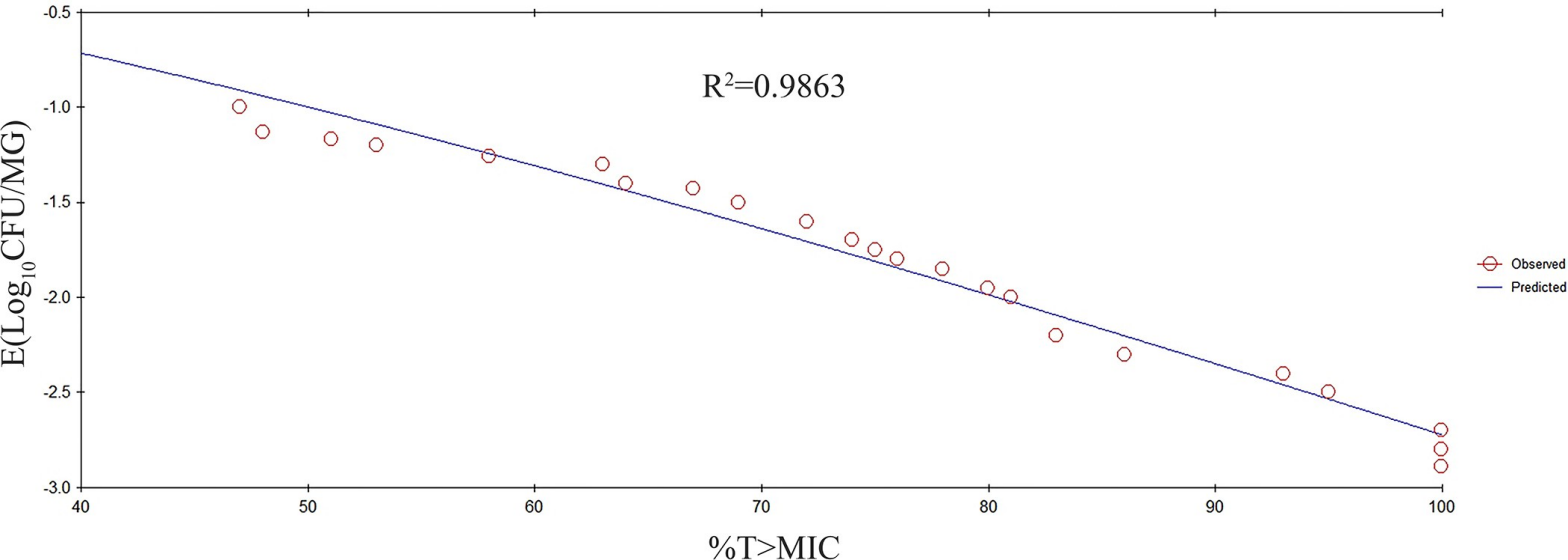
The inhibitory Sigmoid E_max_ relationship between *in vivo* antibacterial effects (△log CFU/MG) and PK/PD index of %T>MIC against *S*. *agalactiae* 3–64. The line represents the fit of the model to the data. The circles represent the observed PD data. R^2^ indicates the correlation coefficient.

**Fig 6:**
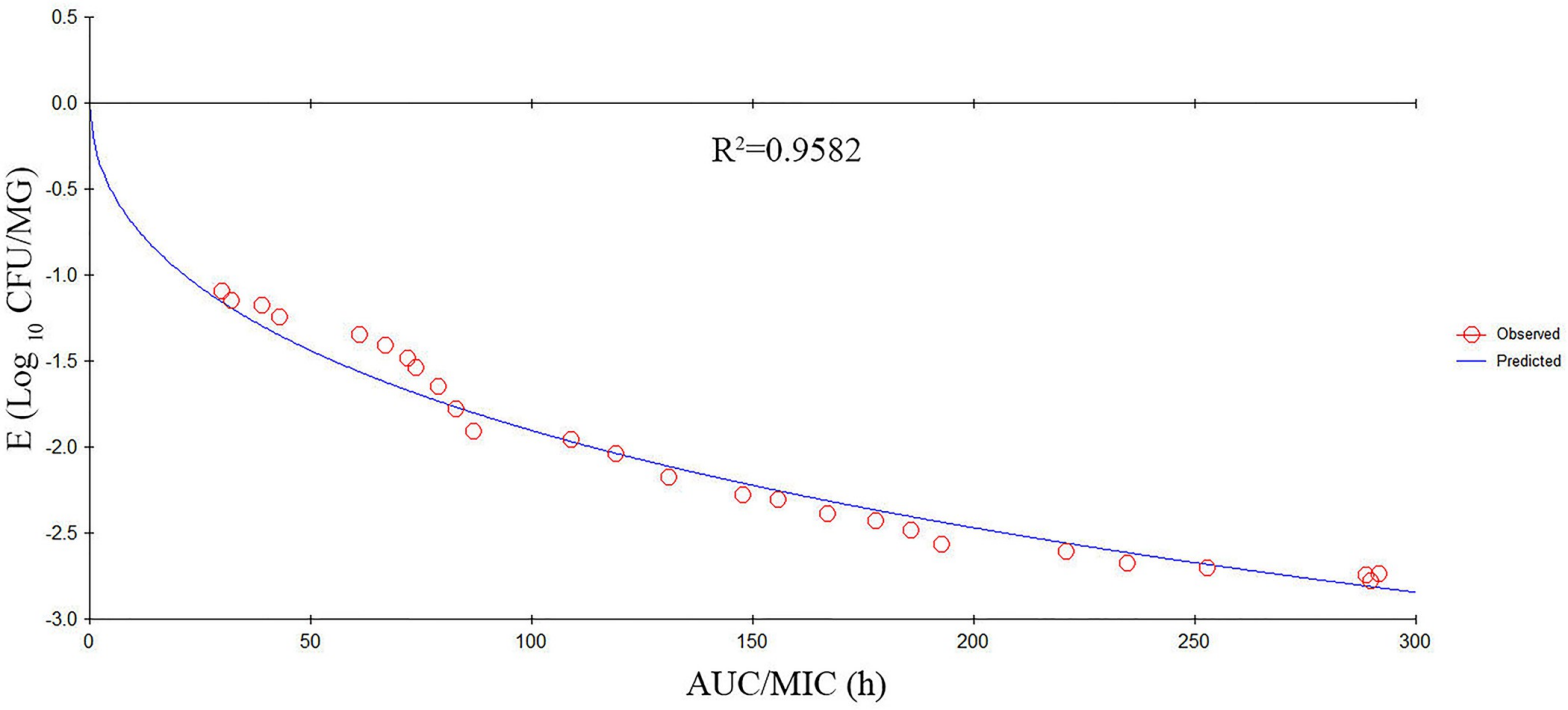
The inhibitory Sigmoid E_max_ relationship between *in vivo* antibacterial effects (△log CFU/MG) and the PK/PD index of AUC/MIC against *S*. *agalactiae* 3–64. The line represents the fit of the model to the data. The circles represent the observed PD data. R^2^ indicates the correlation coefficient.

**Fig 7:**
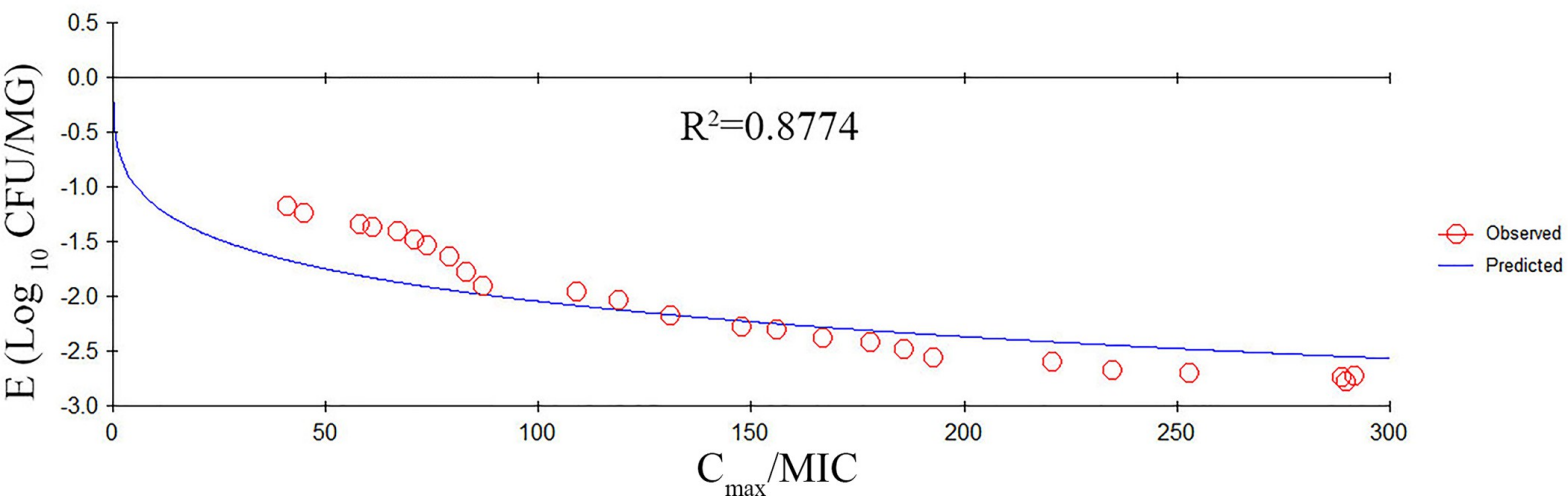
The inhibitory Sigmoid E_max_ relationship between *in vivo* antibacterial effects (△log CFU/MG) and the PK/PD index of C_max_/MIC against *S*. *agalactiae* 3–64. The line represents the fit of the model to the data. The circles represent the observed PD data. R^2^ indicates the correlation coefficient.

PK/PD integration of the various PK/PD indexes *versus* the antibacterial effectiveness for the inhibitory sigmoid E_max_ model are shown in Figs 5–7. The R^2^ values between the observed PD and predicted PD data of %T > MIC, AUC/MIC, and C_max_/MIC were 0.9863, 0.9582, and 0.8774, respectively. The key PK/PD parameters are summarized in Table 3. The target values of the PK/PD indexes to produce a 0.5-log_10_ CFU/MG reduction, 1-log_10_ CFU/MG reduction, and 2-log_10_ CFU/MG reduction were 31%, 47%, and 81% for %T > MIC; 39 h, 79 h, and 101 h for AUC/MIC; and 43, 98, and 147 for C_max_/MIC, respectively.

**Table 3:** The key PK/PD parameters for %T>MIC to achieve different antibacterial effects.

Parameter	Values
E_max_ (log_10_CFU/MG)	1.12
E_0_ (log_10_ CFU/MG)	-2.89
EC_50_ (h)	44.79
N	3.26
%T>MIC for 0.5-log_10_ reduction	31
%T>MIC for 1-log_10_ reduction	47
%T>MIC for 2-log_10_ reduction	81

E_max_ represents the △logCFU72 h in the drug-free control samples; E_0_ indicates the △logCFU72 h in experimental samples containing cefquinome when reached the maximum antibacterial effect; EC_50_ denotes the PK-PD indexes for the drug that means 50% of the maximum antibacterial effect, and N indicates the Hill coefficient (indicating effect curve steepness estimates for PK-PD indexes).

## Discussion

In veterinary drug research, PK and PD data are often established in separate parallel studies to formulate the drug delivery scheme which is evaluated and verified in subsequent clinical trials [35]. However, with the widespread use of antibiotics, bacterial resistance has gradually emerged. PK/PD modelling is a vital approach to optimise the use of antibacterial drugs. The elimination half-life (T1/2e) identified in this study (0.49±0.083 hrs) was similar to that previously reported i.e. 0.4 hrs for intramammary administration in an experimental mouse model of *S*. *aureus* mastitis and 0.43 hrs for the intramuscular injection in the black swan model [27, 36]. However, the value was significantly lower than reported for intramammary administration in lactating Chinese dairy cows (4.63 hrs) which detected the drug concentration in milk and for intramammary administration in an *Escherichiacoli* lactating mouse mastitis model (12.63 hrs) that detected the drug concentration in the MGs [29, 37]. Compared to the cefquinome concentration in MGs, the cefquinome concentration in plasma was much lower which also occurs in cows. This may be related to the chemical properties of cefquinome.

Cefquinome is an organic acid with low fat solubility and pKa values of 2.51 and 2.91 [38]. This causes the distribution of cefquinome to be less extensive and so it cannot penetrate membranes and cross the blood-MG barrier, preventing the drug from reaching the blood from the MG. The T_1/2α_ of 0.07±0.05 hrs was identical to the 0.07 hrs previously reported [36]. In addition to oral administration, the body can rapidly absorb other forms of cefquinome. After metabolism *in vivo*, cefquinome is mainly excreted in the urine through the kidneys [38].

In the present study, after 9 hrs of inoculation in mice MGs, the *S*. *agalactiae* bacterial burden reached approximately 10^7^ CFU/MG. These data showed that the *S*. *agalactiae*-induced mastitis model could sufficiently replicate acute mastitis for bacterial evaluations. Low and high-concentration groups were designed to observe the bactericidal effect of cefquinome on *S*. *agalactiae*. The low-concentration group achieved bactericidal efficacy when the drug concentration was >4 × MIC. However, for the high-concentration group, cefquinome achieved only a bacteriostatic effect. These data agree with a previous report [39]. Significant differences were observed between the low-concentration and high-concentration groups (*p<*0.05) that may be related to the fact that cefquinome is a beta-lactam which is a bactericidal drug at the exponentialstage of bacteria.

Penicillin-binding proteins (PBPs) are needed for the survival, growth, and reproduction of bacteria. PBPs are also the binding sites for beta-lactam antibiotics [40] which cause bacterial death by creating defects in bacterial cell walls [41]. In this study, based on the bacterial killing curves, the low-concentration group had a higher growth rate (more bacteria were present at the exponential stage) compared to the high-concentration group. The greater number of exponential growing bacteria means that more PBPs could combine with the cefquinome. Hence, the bactericidal effect in the low-concentration group was greater than that in the high-concentration group.

Different bacteria have been used to describe the association between PK/PD indexes and cefquinome antibacterial activity in different animal infection models. A prior report used *Haemophilus parasuis* to study the antibacterial activity of cefquinome. The data suggested that the %T > MIC required for 3-log_10_ drop and 4-log_10_ drop were 61% and 71%, respectively [42]. A later study investigated the effects of cefquinome on *Actinobacillus pleuropneumoniae* using a piglet tissue cage model and reported that %T > MIC achieved 11.59%, 27.49%, and 59.81% with respective 1/3-log_10_, 2/3-log_10_, and 1-log_10_ reductions [43]. The same group also used *Escherichia coli* to examine cefquinome antibacterial efficacy and calculated that the values of %T > MIC to achieve 1/6-log_10_ reductions, 1/3-log_10_ reductions, and 1/2-log_10_ reductions were 3.97%, 17.08%, and 52.68%, respectively [44]. The efficacy of cefquinome was also reported for *Klebsiella pneumonia* and *Staphylococcus aureus* in an *ex vivo* dog model and an *in vivo* rabbit tissue cage infection model [21, 45]. All these studies demonstrated that cefquinome had effective antimicrobial activity against these pathogens. However, to the best of our knowledge, no previous study has reported on the efficacy of cefquinome against *S*. *agalactiae*.

In the current study, we used an experimental *S*. *agalactiae* mastitis model system to investigate the interactions between the PK/PD indexes and cefquinome activity against *S*. *agalactiae*. The %T>MIC was the PK/PD index that most effectively described the antibacterial activity of cefquinome against *S*. *agalactiae*. When the *in vivo* %T > MIC values were 31%, 47%, and 81%, there were 0.5-log_10_ units, 1-log_10_ units, 2-log_10_ units reductions observed, respectively. Studies have shown that %T > MIC is a vital index for describing the PK/PD relationship of the cefquinome concerning bactericidal activity [46, 47]. Here, the correlation coefficient (R^2^) values of %T > MIC, AUC/MIC, and C_max_/MIC were 0.9863, 0.9582, and 0.8774, respectively. AUC/MIC has been used to describe the relationship between PK and PD for concentration-dependent drugs. However, in this study, the R^2^ of %T > MIC and AUC/MIC were very close showing that both indexes were useful in this model. These data are in agreement with a previous report by Yu et al. [28]. The cefquinome concentrations in the blood and MG were not identical due to the blood-milk barrier which explains this result.

In this study, we showed that specific doses of cefquinome cause different therapeutic impacts on the *S*. *agalactiae*-induced mastitis model. We also demonstrated that cefquinome can cause a reduction of 2.8-log_10_ CFU/MG in the *in vivo* killing-time curve to achieve a bactericidal effect *in vivo*.

## Supporting information

S1 Fig Chromatograms of blank plasma sample, standard cefquinome solution sample and sample solution sample. **Together with standard curve.** The samples were detected by LC-ESI-MS/MS as described in the method section.
(TIF)

S1 Table *In vitro* cefquinome killing curve against *S*. *agalactiae* 3–64. Antibacterial effects begin at an initial inoculum of 10^6^ CFU/mL.
(DOC)

S2 Table *In vitro* cefquinome killing curve against *S*. *agalactiae* 3–64. Antibacterial effects begin at an initial inoculum of 10^7^ CFU/mL.
(DOC)

S3 Table *In vivo* cefquinome PD studies in a murine mastitis model. Testing dosage regimens were a single dose of 30 μg/MG, 60 μg/MG, 120 μg/MG, and 240 μg/MG by intramammary administration.
(DOCX)

S4 Table I*n vivo* antibacterial effects (△log CFU/MG) *versus* PK/PD index of %T>MIC against *S*. *agalactiae* 3–64. (DOC)

S5 Table I*n vivo* antibacterial effects (△log CFU/MG) *versus* PK/PD index of AUC/MIC against *S*. *agalactiae* 3–64. (DOCX)

S6 Table I*n vivo* antibacterial effects (△log CFU/MG) *versus* PK/PD index of C_max_/MIC against *S*. *agalactiae* 3–64. (DOC)

## Abbreviations

AUC: The area under the concentration-time curve
MIC: Minimum inhibitory concentration
AUC/MIC: The area under the concentration-time curve to MIC ratio
C_max_: The peak concentration
C_max_/MIC: The peak concentration divided by the MIC
MBC: Minimum bactericidal concentration
HPLC: High-performance liquid chromatography
MS: Mass spectrometry
ESI: Electron spray ionisation
PK/PD: Pharmacokinetic/pharmacodynamic
T_max_: The time to achieve maximum concentration
T_1/2e_: Elimination half-life
%T>MIC: The percentage of concentration that excels MIC
