# Supplementary figures and images for: The pharmacokinetics and pharmacodynamics of cefquinome against Streptococcus agalactiae in a murine mastitis model
Source: PLoS One. 2023 Jan 25;18(1):e0278306. doi: 10.1371/journal.pone.0278306 (PMC9876276; doi:10.1371/journal.pone.0278306)

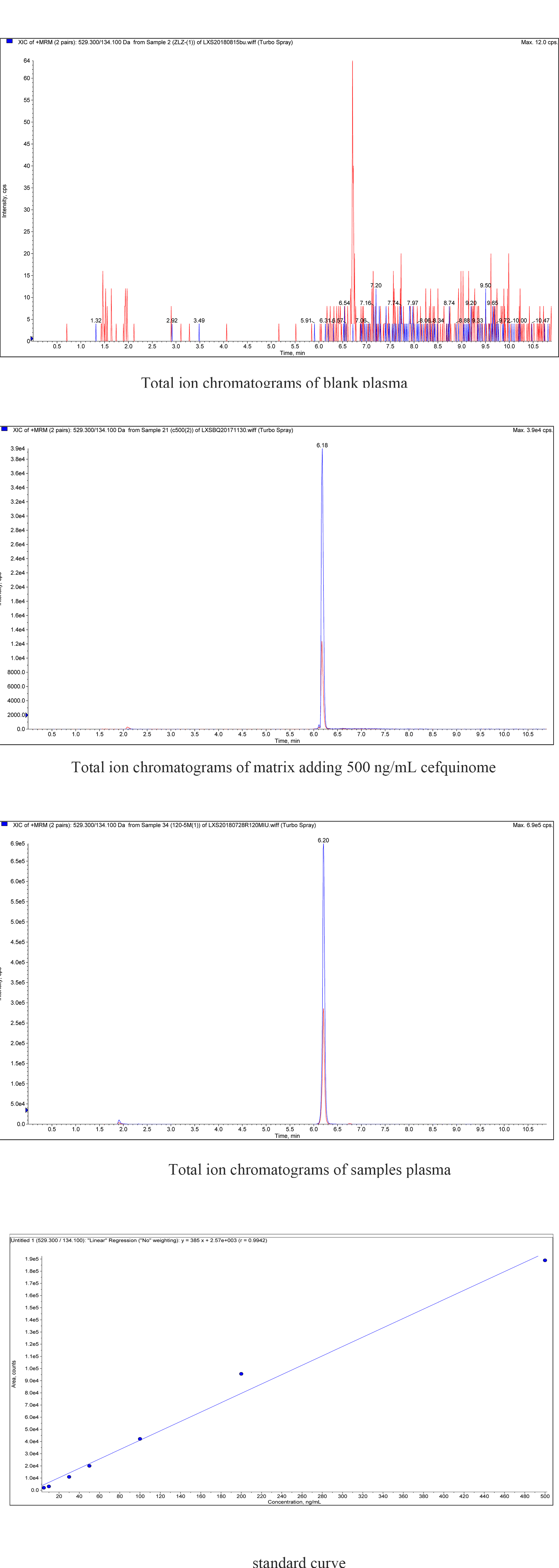

Supplement: S1 Fig — Together with standard curve. The samples were detected by LC-ESI-MS/MS as described in the method section. (TIF) [file pone.0278306.s001.tif]
