# Supplementary material for: The pharmacokinetics and pharmacodynamics of cefquinome against Streptococcus agalactiae in a murine mastitis model
Source: PLoS One. 2023 Jan 25;18(1):e0278306. doi: 10.1371/journal.pone.0278306 (PMC9876276; doi:10.1371/journal.pone.0278306)
Supplement: S1 Table — Antibacterial effects begin at an initial inoculum of 106 CFU/mL. (DOC) [file pone.0278306.s002.doc]

**The pharmacokinetics and pharmacodynamics of Cefquinome against *Streptococcus agalactiae* in a** **Murine Mastitis Model**

Qingwen Yang1, Chenghuan Zhang2, Xuesong Liu3,4, Longfei Zhang5, , KangYong1, Qian Lv1, Yi Zhang1, Liang Chen3, Peng Zhong3,4, Yun Liu2*

| Time (h) | the density of the *Streptococcus agalactiae* (log_10_CFU/MG) | | | | | | |
| --- | --- | --- | --- | --- | --- | --- | --- |
|  | Control | 0.5×MIC | 1×MIC | 2×MIC | 4×MIC | 8×MIC | 16×MIC |
| 3 | 6.00 | 6.00 | 6.00 | 6.00 | 6.00 | 6.00 | 6.00 |
| 6 | 6.77 | 5.94 | 5.56 | 5.07 | 3.98 | 3.94 | 3.91 |
| 9 | 7.41 | 5.89 | 5.49 | 5.00 | 3.83 | 3.74 | 3.69 |
| 12 | 7.56 | 5.85 | 5.36 | 4.85 | 3.73 | 3.69 | 3.65 |

**S1 Table. *In vitro* cefquinome killing curve against *S*. *agalactiae* 3-64.** Antibacterial effects begin at an initial inoculum of 10^6^ CFU/mL.
