# Supplementary material for: The pharmacokinetics and pharmacodynamics of cefquinome against Streptococcus agalactiae in a murine mastitis model
Source: PLoS One. 2023 Jan 25;18(1):e0278306. doi: 10.1371/journal.pone.0278306 (PMC9876276; doi:10.1371/journal.pone.0278306)
Supplement: S2 Table — Antibacterial effects begin at an initial inoculum of 107 CFU/mL. (DOC) [file pone.0278306.s003.doc]

**The pharmacokinetics and pharmacodynamics of Cefquinome against *Streptococcus agalactiae* in a** **Murine Mastitis Model**

Qingwen Yang^1^, Chenghuan Zhang^2^, Xuesong Liu^3,4^, Longfei Zhang^5^, , KangYong^1^, Qian Lv^1^, Yi Zhang^1^, Liang Chen^3^, Peng Zhong^3,4^, Yun Liu^2^*

**S2 Table. *In vitro* cefquinome killing curve against *S*. *agalactiae* 3-64.** Antibacterial effects begin at an initial inoculum of 10^7^ CFU/mL.

| Time (h) | the density of the *Streptococcus agalactiae* (log_10_CFU/MG) | | | | | | |
| --- | --- | --- | --- | --- | --- | --- | --- |
|  | Control | 0.5×MIC | 1×MIC | 2×MIC | 4×MIC | 8×MIC | 16×MIC |
| 3 | 7.00 | 7.00 | 7.00 | 7.00 | 7.00 | 7.00 | 7.00 |
| 6 | 7.43 | 6.94 | 6.56 | 6.20 | 5.98 | 5.94 | 5.82 |
| 9 | 7.53 | 6.89 | 6.49 | 6.07 | 5.83 | 5.74 | 5.66 |
| 12 | 7.61 | 6.85 | 6.36 | 5.93 | 5.73 | 5.69 | 5.46 |
