# Supplementary material for: The pharmacokinetics and pharmacodynamics of cefquinome against Streptococcus agalactiae in a murine mastitis model
Source: PLoS One. 2023 Jan 25;18(1):e0278306. doi: 10.1371/journal.pone.0278306 (PMC9876276; doi:10.1371/journal.pone.0278306)
Supplement: S3 Table — Testing dosage regimens were a single dose of 30 μg/MG, 60 μg/MG, 120 μg/MG, and 240 μg/MG by intramammary administration. (DOCX) [file pone.0278306.s004.docx]

**The pharmacokinetics and pharmacodynamics of Cefquinome against *Streptococcus agalactiae* in a** **Murine Mastitis Model**

Qingwen Yang^1^, Chenghuan Zhang^2^, Xuesong Liu^3,4^, Longfei Zhang^5^, , KangYong^1^, Qian Lv^1^, Yi Zhang^1^, Liang Chen^3^, Peng Zhong^3,4^, Yun Liu^2^*

**S2 Tab. *In vivo* cefquinome PD studies in a murine mastitis model.** The change in the log_10_ CFU/MG was measured after 72 hrs of treatment. Changes in the viable cell density (CFU/MG) of *S. agalactiae* and the concentrations of antibiotics (×MIC) *in vivo* following a single treatment with cefquinome. Testing dosage regimens were a single dose of 30 μg/MG, 60 μg/MG, 120 μg/MG, and 240 μg/MG by intramammary administration.

| Time (h) | the density of the *Streptococcus agalactiae* (log_10_CFU/MG) | | | | |
| --- | --- | --- | --- | --- | --- |
|  | Control | 30 μg/MG | 60 μg/MG | 120 μg/MG | 240 μg/MG |
| 0 | 7.00 | 7.00 | 7.00 | 7.00 | 7.00 |
| 3 | 7.14 | 6.93 | 6.78 | 6.65 | 6.61 |
| 6 | 7.17 | 6.85 | 6.74 | 6.44 | 6.34 |
| 9 | 7.20 | 6.82 | 6.62 | 6.32 | 6.07 |
| 12 | 7.24 | 6.74 | 6.46 | 6.04 | 5.97 |
| 24 | 7.25 | 6.66 | 6.27 | 5.82 | 5.70 |
| 48 | 7.23 | 6.62 | 6.04 | 5.49 | 5.47 |
| 72 | 7.25 | 6.56 | 5.97 | 4.93 | 4.81 |
