# Supplementary material for: The pharmacokinetics and pharmacodynamics of cefquinome against Streptococcus agalactiae in a murine mastitis model
Source: PLoS One. 2023 Jan 25;18(1):e0278306. doi: 10.1371/journal.pone.0278306 (PMC9876276; doi:10.1371/journal.pone.0278306)
Supplement: S4 Table — (DOC) [file pone.0278306.s005.doc]

**The pharmacokinetics and pharmacodynamics of Cefquinome against *Streptococcus agalactiae* in a** **Murine Mastitis Model**

Qingwen Yang^1^, Chenghuan Zhang^2^, Xuesong Liu^3,4^, Longfei Zhang^5^, , KangYong^1^, Qian Lv^1^, Yi Zhang^1^, Liang Chen^3^, Peng Zhong^3,4^, Yun Liu^2^*

S3 Table. I*n vivo* antibacterial effects (△log CFU/MG ) *versus* PK/PD index of %T>MIC against *S. agalactiae* 3-64.

| I*n vivo* antibacterial effects  (△log CFU/MG ) | %T>MIC |
| --- | --- |
| -1 | 47 |
| -1.13 | 48 |
| -1.14 | 49 |
| -1.16 | 50 |
| -1.2 | 52 |
| -1.3 | 54 |
| -1.35 | 56 |
| -1.4 | 59 |
| -1.46 | 61 |
| -1.5 | 64 |
| -1.6 | 66 |
| -1.7 | 69 |
| -1.75 | 71 |
| -1.8 | 73 |
| -1.82 | 75 |
| -1.86 | 76 |
| -1.88 | 78 |
| -1.95 | 79 |
| -2 | 82 |
| -2.2 | 86 |
| -2.3 | 93 |
| -2.5 | 95 |
| -2.7 | 100 |
| -2.73 | 100 |
| -2.89 | 100 |
