# Supplementary material for: The pharmacokinetics and pharmacodynamics of cefquinome against Streptococcus agalactiae in a murine mastitis model
Source: PLoS One. 2023 Jan 25;18(1):e0278306. doi: 10.1371/journal.pone.0278306 (PMC9876276; doi:10.1371/journal.pone.0278306)
Supplement: S5 Table — (DOCX) [file pone.0278306.s006.docx]

**The pharmacokinetics and pharmacodynamics of Cefquinome against *Streptococcus agalactiae* in a** **Murine Mastitis Model**

Qingwen Yang^1^, Chenghuan Zhang^2^, Xuesong Liu^3,4^, Longfei Zhang^5^, , KangYong^1^, Qian Lv^1^, Yi Zhang^1^, Liang Chen^3^, Peng Zhong^3,4^, Yun Liu^2^*

**S4 Table.** I*n vivo* antibacterial effects (△log CFU/MG ) *versus* PK/PD index of AUC/MIC against *S. agalactiae* 3-64.

| I*n vivo* antibacterial effects  (△log CFU/MG ) | AUC/MIC |
| --- | --- |
| -1 | 43 |
| -1.13 | 47 |
| -1.14 | 49 |
| -1.16 | 53 |
| -1.2 | 55 |
| -1.3 | 56 |
| -1.35 | 58 |
| -1.4 | 62 |
| -1.46 | 64 |
| -1.5 | 68 |
| -1.6 | 73 |
| -1.7 | 79 |
| -1.75 | 87 |
| -1.8 | 89 |
| -1.82 | 93 |
| -1.86 | 105 |
| -1.88 | 109 |
| -1.95 | 115 |
| -2 | 126 |
| -2.2 | 156 |
| -2.3 | 182 |
| -2.5 | 265 |
| -2.7 | 282 |
| -2.73 | 285 |
| -2.89 | 289 |
